# Evaluating sleep quality in a non-intrusive manner using contactless ballistocardiography and audio signals through a LSTM-TCN machine learning model

**DOI:** 10.3389/fnetp.2026.1779111

**Published:** 2026-05-12

**Authors:** Dominic Jaworski, Tae-Ho Kim, Bohyung Choi, Edward J. Park

**Affiliations:** 1 Biomechatronic Systems Laboratory, School of Mechatronic Systems Engineering, Simon Fraser University, Surrey, BC, Canada; 2 WearTech Labs, Simon Fraser University, City Centre 2, Surrey, BC, Canada

**Keywords:** audio, ballistocardiography, cardiorespiratory, network physiology, sensors, sleep

## Abstract

**Background:**

Non-intrusive sleep detection devices are increasingly sought after because conventional sleep studies require multiple body-attached sensors, which are uncomfortable and impractical for routine use. Integrating sensors and devices into a system that measures a network of physiological signals and their interactions (e.g., cardiorespiratory coupling) non-intrusively can help analyze sleep quality without negatively affecting natural sleep. Effectively measuring natural sleep quality can provide awareness that helps individuals adjust daily habits to improve the amount of good-quality sleep and support earlier identification of sleep disorders without worsening them with the uncomfortable polysomnography (PSG) setup.

**Methods:**

In this study, we introduce a diagnostic tool that combines a ballistocardiogram (BCG) and a microphone as a potential substitute for conventional PSG methods for collecting cardiorespiratory signals while participants sleep. The cardiorespiratory signals were processed in the time, frequency, and nonlinear domains, with an emphasis on nonlinear analysis because of the dynamic nature of physiological processes linked to the nervous system. Also, cardiopulmonary coupling (CPC) was measured to observe the relationship between cardiac and respiratory activity, consistent with network physiology perspectives on coupled subsystem dynamics, which would help the model classify sleep stages. Furthermore, the audio signals were processed through spectral and autocorrelation methods to obtain respiratory activity from sleep sounds.

**Results:**

These features were used to train a model combining long short-term memory (LSTM) and a temporal convolutional network (TCN), achieving an accuracy of 80.51% (Cohen’s κ = 0.65) for wake/non-REM/REM sleep stages compared with PSG under leave-one-subject-out cross-validation.

**Conclusion:**

A non-intrusive system for evaluating sleep stages can enable medical professionals to diagnose sleep disorders without negatively affecting physiological data from patients using PSG-based studies. By quantifying cardiorespiratory interactions from contactless sensing, this approach provides a network physiology–aligned framework for longitudinal, in-home monitoring of sleep-related subsystem dynamics.

## 1 Introduction

Polysomnography (PSG) has been widely employed as a standard tool for evaluating sleep quality and diagnosing sleep disorders, measuring multiple physiological signals to assess sleep status. However, PSG is intrusive, often resulting in discomfort and making it impractical for personal use in an in-home environment (Chinoy et al., 2021). Consequently, more convenient options for evaluating sleep quality have been explored using wearable and non-contact devices that do not disrupt the patient’s sleep (Chinoy et al., 2021). Ballistocardiography (BCG) assesses cardiorespiratory parameters using sensitive accelerometers that detect mattress vibrations produced by the body’s recoil forces from the heartbeat and blood flow through the heart (Uguz et al., 2020; Mora et al., 2020). BCG can measure cardiac parameters, similar to an electrocardiogram (ECG), but with a different sensing mechanism. Also, it can extract features such as heart rate variability (HRV) during sleep without direct contact or interruption (Shin et al., 2011). Changes in HRV reflect activity in the autonomic nervous system (ANS), which can correlate with the body’s restful or active state and serve as an indicator of sleep quality (Tobaldini et al., 2013). Adaptations in the ANS can signal transitions among sleep stages or reveal potential sleep disorders if they fail to adjust to environmental changes.

The intrusive nature of PSG may lead to an unnatural sleep cycle and poor diagnosis for sleep disorders (Sazonov et al., 2004). Therefore, using sensors and devices that enable non-intrusive evaluation of sleep quality would allow one to sleep comfortably while their natural physiological signals are analyzed during sleep. Audio recordings through a non-intrusive microphone during sleep can capture respiration, snoring, movement, and external sounds, all of which can contribute to sleep stage evaluations (Dafna et al., 2015). Furthermore, various personal devices, such as smartphones and wireless earphones, already feature high-quality microphones and are generally designed to be more accessible and cost-effective than clinical devices, enabling users to track trends in their health frequently (Mathunjwa et al., 2025; Lee et al., 2019). Although such devices may not provide clinically precise health measurements, deviations from normal patterns can prompt users to seek a professional medical evaluation to prevent any sleep disorders or health complications before they occur.

Analyzing sleep quality using clinical devices, such as PSG, typically requires manual scoring by trained personnel who interpret each signal according to established rules and guidelines (Berry et al., 2020). However, interrater reliability is about 0.76 among trained personnel, indicating that professionals still differ in their interpretation of sleep stages from clinical equipment (Lee et al., 2022). Therefore, there is a wide range of efforts to advance wearable devices equipped with sophisticated sensors and relevant software, enabling sleep detection without specialized sleep-scoring expertise (de Zambotti et al., 2019). Consumer wearables (e.g., smartwatches and fitness trackers) provide health and sleep information in understandable applications that can be linked to users’ phones. Beyond wearable technologies, contactless consumer devices placed on the mattress, such as piezoelectric sensors for measuring cardiorespiratory and movement activity, as well as bedside devices that assess these parameters using low-power radio-frequency waves, have been explored (Chinoy et al., 2021). The convenient nature of these devices allows them to be used for extended periods, enabling a user to monitor their sleep and health patterns and receive awareness of any significant changes. Across these modalities, state-of-the-art sleep staging pipelines typically apply modality-specific preprocessing to cardiac activity, including filtering to isolate cardiac components, signal-quality or motion-artifact screening, peak/beat detection to estimate inter-beat intervals (IBIs), and subsequent HR/HRV feature computation with appropriate normalization and outlier handling (Uguz et al., 2020; Mora et al., 2020). We follow the same general structure for BCG-derived cardiac signals in this work (filtering and artifact handling prior to HRV feature extraction), and we note that preprocessing choices can substantially affect downstream staging performance. These devices can measure physiological parameters, such as heart and respiratory rates, which can serve as features for training machine learning algorithms to classify sleep stages (Yoon and Choi, 2023). Cardiac activity measured by these sensors enables the analysis of HRV changes during sleep, reflecting transitions between restful and active states. Time-domain, frequency-domain, and nonlinear HRV analyses capture complementary aspects of cardiac autonomic regulation and are commonly used as features for sleep stage classification (Shaffer and Ginsberg, 2017). Machine learning models that can account for sleep cycles’ temporal order, such as the sequence from lying down and resting, to the middle portion for sleep and waking, and finally getting out of bed—would be able to classify sleep stages as they transition from lighter to deeper stages. For example, a long short-term memory (LSTM) model can store long-term dependencies from a time-series signal and generate classifications of the present based on the last short-term output and the long-term memory state (Rahda et al., 2019). Another model that considers the temporal order of time series is the temporal convolutional network (TCN), which suits sequential data through causal and dilated convolutions that compute present and past values and increase the size of the receptive field without increasing the number of parameters, thereby efficiently capturing long-term data (Ismail et al., 2023). Both the LSTM network and TCN have shown good performance in the literature, achieving accuracy of 80.75% and 86.3%, respectively, for sleep stage classification when applied to physiological data from ECG and PSG devices (Zhang et al., 2019; Jung and Chung, 2024). Selecting appropriate physiological features to train deep learning models can effectively evaluate sleep stages and be applied to wearable and contactless devices and clinical settings to support user awareness of sleep health.

In this study, we measured cardiorespiratory interactions as network physiology parameters using an Internet of Things (IoT) system including a contactless BCG device and an audio microphone to evaluate sleep quality in a non-intrusive environment. The devices were evaluated in a BCG + audio (contactless core system) where features from the included devices were combined within the contactless core system. Cardiac activity measured using BCG and respiratory activity derived from microphone audio enabled the assessment of interactions between the cardiac and respiratory systems as a physiological network across sleep-stage transitions. Furthermore, the methodology and signal processing in this work builds on our previous publications that used machine learning methods with a focus on nonlinear analysis of cardiorespiratory parameters to evaluate sleep stages (Jaworski and Park, 2025; Jaworski and Park, 2024).

## 2 Materials and methods

### 2.1 Experiment protocol

**FIGURE 1:**
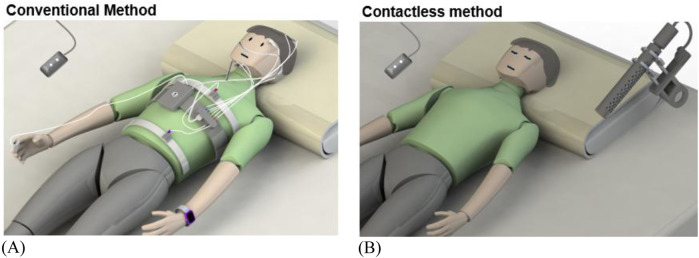
Conventional sleep study compared to the proposed comfortable sleep study method. **(A)** Shows a participant undergoing PSG system with cables and electrodes attached to the body, while **(B)** shows the proposed setup, with devices placed around the bedroom without direct contact.

This study involved human participants and was approved by Simon Fraser University’s research ethics board (2017062) to ensure ethical and safety standards were maintained during the experiments. All participants provided informed consent prior to the commencement of the sleep studies and were briefed on the protocol. Nine healthy adults (5 female and 4 male) with an average BMI and aged 22–40 years participated in this study. Each participant completed three sleep cycles, each lasting at least 3 h, for a total of 27 sleep cycles and approximately 90 h of total recorded sleep time. Multiple sleep sessions were conducted to observe trends in participant sleep and to allow them to acclimate to the PSG system throughout the experiment. Sleeping with a PSG system can induce a “first night effect,” in which participants experience poor sleep due to the wires and electrodes attached to their bodies and heads, leading to analyses that do not accurately reflect their natural sleep (Herbst et al., 2010). Figure 1 illustrates a conventional clinical sleep study, which uses a PSG system with all the wires and electrodes placed on the user’s body, compared with the proposed system, which uses two non-intrusive devices placed in and around the bed to allow for comfortable, natural sleep while measuring physiological parameters.

**FIGURE 2:**
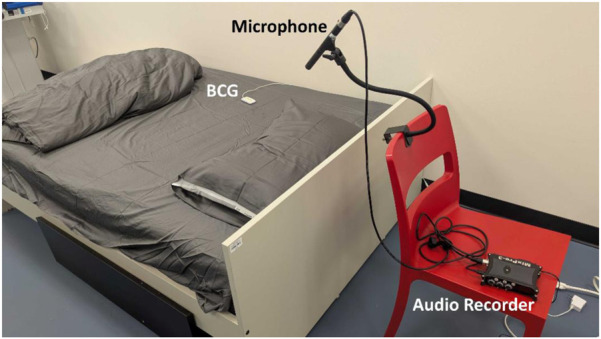
Experimental bedroom setup for the sleep study. The BCG was placed on top of the mattress towards the right side of the bed, closer to the wall to prevent the BCG from moving when participants entered and exited the bed. The BCG sensor measures mattress vibrations as acceleration (via an embedded accelerometer), from which cardiac and respiratory activity are derived. The microphone was placed at the top of the bed and directed towards the pillow area to measure the participant’s respiration.

To simulate an in-home environment, participants slept in a full-sized bed. The room was kept at a constant 21 °C, and participants were permitted to use as few covers as desired for comfort during the experimental sessions. The BCG sensor was placed on the mattress and secured by a magnetic clamp on the bed sheet lining to prevent movement during the measurement of cardiorespiratory signals. The BCG device streamed data in real time via Wi-Fi to a laptop running a message queuing telemetry transport (MQTT) server, which stored the data in a structured query language (SQL) database monitored by the research team. The BCG and IoT sleep monitoring system is presented in more detail in our previous publication (Jaworski et al., 2021). A high-quality directional microphone was placed about half a meter above the bed and directed toward the participant’s head. A PSG system was used as a reference for the sleep quality analysis compared to the developed algorithm. The experimental bedroom setup in our lab, with the BCG, microphone, and audio recorder around the bed, is depicted in Figure 2.

### 2.2 Polysomnography

**FIGURE 3:**
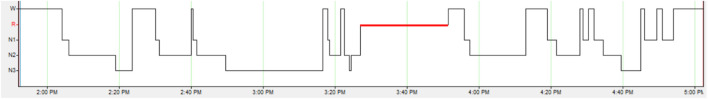
Hypnogram obtained from the PSG RemLogic software showing all scored sleep stages throughout the sleep cycle. Sleep stages include wake, N1, N2, N3, and REM.

The PSG system used in this study was the Natus Embletta X100 by Embla Systems Inc. (Bloomfield, CO, United States), which consisted of an electroencephalogram (EEG), electrocardiogram (ECG), electrooculogram (EOG), electromyogram (EMG), photoplethysmogram (PPG), respiration bands, and a nasal cannula. The PSG served as the gold standard for the sleep stage classification obtained from the IoT sleep system and devices. The data were downloaded into the RemLogic software provided by Natus and were then visually scored into each sleep stage (Wake, N1, N2, N3, rapid eye movement (REM)) using the rules and guidelines established by the American Academy of Sleep Medicine (AASM) (Berry et al., 2020). A hypnogram exported from the software, showing all sleep stages for one sleep cycle, is shown in Figure 3.

### 2.3 Experimental device sensors

#### 2.3.1 Ballistocardiography

The BCG device used in this study was the Murata SCA11h bed sensor, which measures various cardiorespiratory signals, including heart rate, respiratory rate, heart stroke volume, HRV, and beat-to-beat interval time. The raw acceleration data were sampled at 1 kHz, and the processed cardiorespiratory data were reported at 1 Hz over a wireless network using Murata’s ultralow-noise accelerometers (Meriheina, 2019). The BCG sensor was placed on the mattress to measure vibrations from the body’s subtle recoil forces associated with breathing and cardiac activity (Mora et al., 2020). In contrast, an ECG measures electrical impulses from electrodes attached to the patient, with the QRS complex and R peak indicating the heartbeat (Pino et al., 2017). BCG cardiac signals are analogous to ECG and are identified by IJK, with the J peak corresponding to the heartbeat (Pino et al., 2017). BCG signals are measured via vibrations; they exhibit lag compared to ECG, and movement can result in noisy signal artifacts that need to be filtered out to obtain accurate heart rate measurements (Pino et al., 2017). Such unwanted signals were removed using infinite impulse response (IIR) filters and the low-pass filter shown in Equation 1 to smooth the signals.

$$y\left(t\right)=\left(1-k\right)\times y\left(t-1\right)+k\times x\left(t\right)$$ (1)

where *k* is the filter constant, *x*(*t*) is the original value from the signal, *y*(*t*) is the filtered value, *y*(*t-1*) is the previous value, and *t* is the time (Nurmi, 2016). The BCG was calibrated before participants began the experiment using the Murata software GUI in accordance with the manufacturer’s documentation (Meriheina, 2019; Note and Calibration, 2017). Clinical validation conducted at the University of Turku Sleep Research Centre, Finland, demonstrated that Murata’s BCG sensor’s cardiac signals closely matched PSG readings (Meriheina, 2019; Nurmi, 2016). Additionally, we validated the cardiorespiratory measures of the Murata BCG device against a clinical PSG and a consumer wearable, demonstrating comparable results in our previous publication (Jaworski and Park, 2023).

#### 2.3.2 Audio microphone

The microphone used for this study was the Rode NTG5 (Sydney, Australia), connected to a Sound Devices MixPre-3 II audio recorder, which was set up as in our previous publication (Jaworski and Park, 2025). The audio recorder input gain was set to 70 dB, and the microphone was sampled at 44.1 kHz. The audio signals were exported and processed in Audacity 3.7.5 using noise-reduction functions to remove ambient background noise during the sleep study, focusing on the participants’ respiratory sounds and activity. To ensure that noise reduction functions did not remove the valuable respiration data, each participant’s audio spectrograms were inspected to verify that the breaths were maintained after removing the background noise. Additionally, the directional microphone was angled and directed towards the participant, so the focus was on sounds from the bed, and any outside sounds were not picked up as prominently.

#### 2.3.3 System device synchronization

**FIGURE 4:**
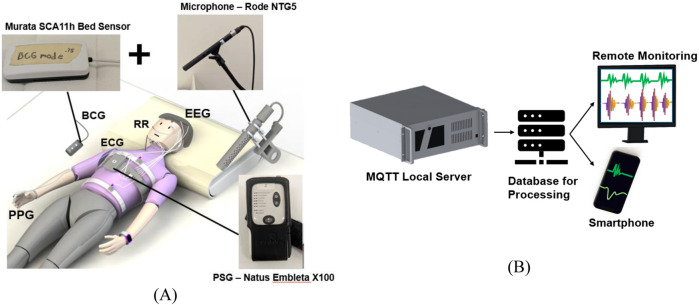
Participant sleeping with each device during the sleep study shown in **(A)** and **(B)**, being the data transfer from sensors through the MQTT broker to a database/ingestion service, with results subsequently made available to clinical or personal devices.

Synchronization across the IoT system was critical for accurate sleep health analysis. The PSG and BCG, have synchronized timestamps with the computers’ clocks, while audio is synchronized by an audible beep from the event button on the PSG. The PSG software creates an event tag that is used to synchronize the audio by removing portions that fall outside the experiment recording time. Participants were instructed to press the event button upon entering or exiting the bed, at the beginning of the experiment, and upon leaving the bed at the end of the experiment. The PSG outputs sleep stages in 30-s epochs, and each device was preprocessed to output results in 30-s intervals to match the PSG reference for model training. The BCG samples were taken at 1 Hz, so the data were windowed into 30-s segments, with lost samples due to network connectivity interpolated to create a complete dataset. Audio signals were separated into 30-s intervals in Audacity and later processed further during the analysis of all signals. Figure 4 presents the schematic of the experimental setup, highlighting each device and the resulting data output in the server for analysis.

### 2.4 Signal processing

#### 2.4.1 Heart rate variability analysis

Cardiorespiratory data were obtained from the BCG and segmented into 30-s epochs to align with the PSG sleep stage output. Additionally, cardiac activity was processed in 5-min overlapping sliding windows, incremented by 30-s intervals, to analyze short-term HRV using many of the metrics and norms established in the literature (Shaffer and Ginsberg, 2017). All the data were processed in MATLAB 2024b, primarily focusing on the cardiorespiratory measurements obtained from each device. The BCG outputs heart rate and respiratory rate in beats and breaths per minute (BPM), as well as the raw J-J peak interval time in milliseconds. Murata’s BCG device also outputs a processed HRV parameter, but it includes respiration depth (Rdepth) in the equation to compensate for excessively high-frequency components associated with respiration events, as shown in Equations 2, 3 (Nurmi, 2016).

$$HRV=\frac{HFHRV}{LFHRV\times Rdepth}$$ (2)

$$Rdepth=\frac{\left|SV-lp\left(SV,k\right)\right|}{SV}$$ (3)

The *R-depth* is calculated by subtracting the sensor’s output absolute stroke volume (SV) from a low-pass-filtered SV using a *k* filtering constant. The *R-depth* is included with the low- and high-frequency HRV ratios (LFHRV and HFHRV, respectively) in milliseconds.

Based on the many metrics and norms regarding HRV, standard features were extracted in the time, frequency, and nonlinear domains. Time domain features such as standard deviation and root mean square of successive JJ intervals (SDNN and RMSSD, respectively); frequency domain features such as low frequency and high frequency HRV and the ratio of LF/HF power; and nonlinear features such as the standard deviations perpendicular to and along the line of identity of a Poincaré plot (SD1 and SD2) and the SD1/SD2 ratio, as well as sample and approximate entropy and detrended fluctuation analysis results (Shaffer and Ginsberg, 2017). More focus was placed on nonlinear domain features, as physiological signals exhibit nonlinear and non-stationary characteristics due to the ANS dynamically adjusting body functions to compensate for external factors, such as rest or activity (Jaworski and Park, 2024; Sadek et al., 2019).

**FIGURE 5:**
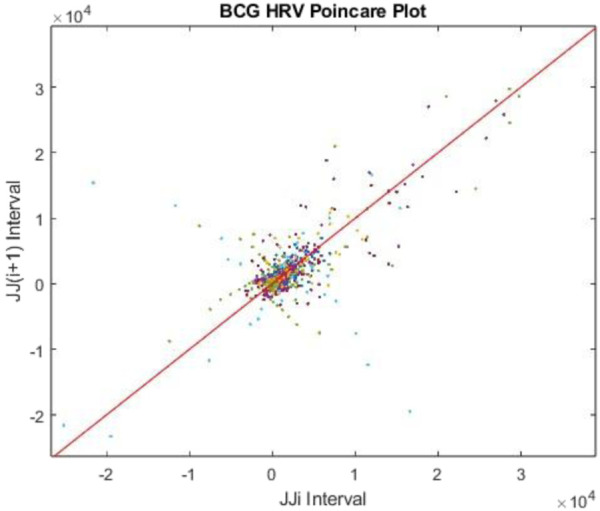
Poincaré plot for consecutive JJ intervals measured from BCG during a sleep cycle.

Poincaré plots are a nonlinear method that maps consecutive JJ intervals and can be related to the balance of the sympathetic (SNS) and parasympathetic (PNS) nervous systems (Equations 4, 5), and their ratio (Shaffer and Ginsberg, 2017; Vollmer, 2015; Behbahani et al., 2013).

$$SD1=\sqrt{\frac{1}{2}\times \sigma \left({JJ}_{i+1}-{JJ}_{i}\right)}$$ (4)

$$SD2=\sqrt{\frac{1}{2}\times \sigma \left({JJ}_{i+1}+{JJ}_{i}\right)}$$ (5)

where *SD*1 and *SD*2 are the width and length of the scatter plot of consecutive JJ intervals, respectively; σ is the variance of the scattered consecutive JJ intervals, indicated by *JJi* and *JJ*_*i+*1_ (Shaffer and Ginsberg, 2017). Additional nonlinear features are extracted from the asymmetric spread of scattered points in a Poincaré plot, represented as a series of asymmetric indices (Khandoker et al., 2013; Yan et al., 2017). The expanded analysis of Poincaré plots is detailed in our previous publication (Jaworski and Park, 2024) and will be briefly summarized here. The line of identity in a Poincaré plot passes through the scatter plot at a 45-degree angle (Figure 5), and each index evaluates the shape of consecutive JJ intervals to identify imbalances between the SNS and PNS (Pawloski et al., 2021). The Porta index simply displays the ratio of how many points are below the line of identity among all the points; the Guzik index measures the ratio of distances of points above the line of identity over all the points; the Ehler index evaluates the skewness of all the points with respect to the line of identity; and finally, the phase and area indexes evaluate the phase angle of all the points above the line of identity and the area produced from the angles to each point (Khandoker et al., 2013; Yan et al., 2017; Pawloski et al., 2021).

**FIGURE 6:**
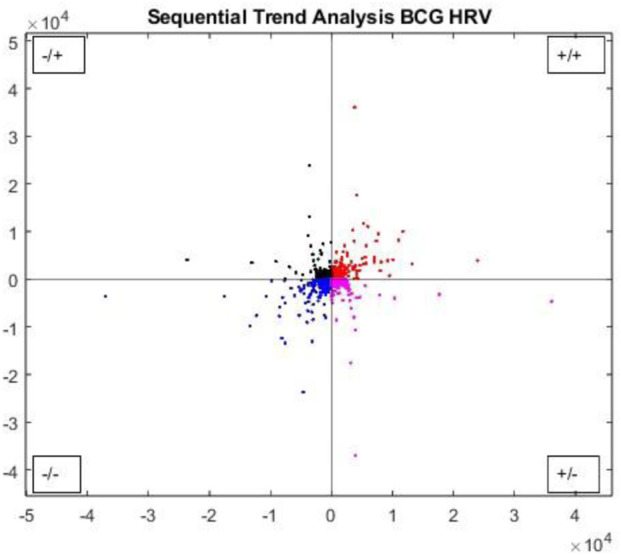
Sequential trend analysis plot for consecutive JJ intervals from BCG during a sleep cycle. Quadrants show increasing/decreasing and acceleration transitions of JJ intervals.

Sequential trend analysis is another nonlinear scatter plot method that plots the difference between successive JJ intervals in quadrants to show instances in which JJ intervals increase or decrease, as shown in Figure 6 (de Carvalho et al., 2003). Increasing values relate to PNS activations, and decreasing values relate to SNS activation; alternating increases and decreases indicate a transition between ANS activations, as evaluated by taking the mean distance of all data points in each quadrant, as in Equation 6 (Srinivas et al., 2007; Srinivas and Reddy, 2010). An example of the STA plot of the JJ interval quadrants is shown in Figure 6.

$$\frac{1}{n-1}\sum_{k=1}^{n-1}\sqrt{{JJ}_{n}^{2}+{JJ}_{n+1}^{2}}$$ (6)

The final nonlinear feature incorporated into this study is Rényi entropy, as it is effective for nonlinear and non-stationary signals like HRV and can be applied to short time series, such as 5-min windows of short-term HRV (Jelinek et al., 2019). Rényi entropy measures complexity in HRV by calculating probabilities using a density-based method that considers subsamples in the vicinity of each sample, i.e., each short time window (Jelinek et al., 2019). Rényi entropy generalizes Shannon entropy in the following Equations 7, 8, 9:

$$H\left(\alpha \right)=\frac{1}{1-\alpha }\log_{2}\left(\sum_{i=1}^{n}\rho_{i}^{\alpha }\right)$$ (7)

$$\rho_{i}=\frac{1}{\sigma \sqrt{2\pi }}\sum_{j=1}^{2}e^{-\frac{{dist}_{ij}^{2}}{2\sigma^{2}}}$$ (8)

$${dist}_{ij}=\sum_{k=0}^{\pi }{\left(x_{i+k}-x_{j+k}\right)}^{2}$$ (9)

with *n* being the number of values, *α* being the order of entropy, the probability from random variables and the Gaussian kernel being *ρ*_*i*_, *σ* being the dispersion, *π* being the number of JJ intervals, and *dist* being the sum of the Euclidean distances of the sequential JJ intervals represented by *x* (Jelinek et al., 2019; Rényi, 1961; Askenazy et al., 2001).

#### 2.4.2 Cardiopulmonary coupling analysis

In addition to evaluating time, frequency, and nonlinear domains, this study incorporated cardiopulmonary coupling (CPC) features that examine the dynamic interactions between heartbeat intervals and the ECG-derived respiration rate (EDR) (Thomas, 2023). CPC was evaluated by calculating the cross-spectral power and coherence between the cardiac RR intervals and amplitude fluctuations of the ECG QRS complexes influenced by respiration. In this study, the heartbeat intervals and EDR will be referred to as JJ intervals, and the BCG-derived respiration rate (BDR) will be used, as BCG was used to evaluate all cardiorespiratory measurements. The method used to evaluate the CPC is based on Thomas et al., 2005 with slight modifications to address the BCG signal sources, which will be explained in the following section. The JJ intervals and BDR are derived from the Murata BCG output signals’ beat-to-beat intervals and respiration rate, which are rescaled to milliseconds and sampled at 1 Hz (Meriheina, 2019). The signals were resampled to 2 Hz by cubic-spline interpolation, windowed into 1024-sample windows, and further split into three 512-subwindow increments of 256 samples each. Within each sub-window, the samples were detrended and processed with a Hanning window before performing a Fourier transform to evaluate the cross-spectral power of both signals. The 1024-sample windows were incremented by 30 s for the entire signals to align with the PSG sleep stage epochs. The Fourier-transformed windows were used to evaluate the cross-spectral power density, as implemented in MATLAB in Equations 10,11, [Disp-formula e11] (Oppenheim et al., 1999; Rabiner and Gold, 1975a; Welch, 1967).

$$P_{xy}\left(\omega \right)=\sum_{m=-\infty }^{\infty }R_{xy}\left(m\right)e^{-j\omega m}$$ (10)

$$R_{xy}\left(m\right)=E\left\{x_{n+m}y_{n}^{*}\right\}=E\left\{x_{n}y_{n-m}^{*}\right\}$$ (11)

*P*_*xy*_ is the cross-power spectral density, where *ω* is the angular frequency and *m* is the time step. *R*_*xy*_ is the cross-correlation sequence of the Fourier transformed RR intervals, and BDR is represented as the complex conjugate, *y*_*n*_ and *x*_*n*,_ respectively. Coherence is evaluated using the magnitude-squared coherence function in MATLAB, as presented in Equation 12 (Welch, 1967; Gómez González et al., 2010; Kay, 1988; Rabiner and Gold, 1975b; Stoica and Moses, 2005).

$$C_{xy}\left(f\right)=\frac{{\left|P_{xy}\left(f\right)\right|}^{2}}{P_{xx}\left(f\right)P_{yy}\left(y\right)}$$ (12)

*C*_*xy*_ is the magnitude squared coherence of the power-spectral density *P*_*xy*_(*f*) divided by the power spectral densities of the RR intervals and BDR presented by *P*_*yy*_ *(f)* and *P*_*xx*_(*f*)*,* respectively. The CPC index is derived from Equation 13.

$$CPC\left(f_{n}\right)={\langle P_{xy}\left(\omega \right)\rangle }^{2}C_{xy}\left(f\right)$$ (13)

where the frequency-averaged cross-spectral power density is combined with the magnitude squared coherence (Thomas et al., 2005). The low, high, and ratio of the frequency components are extracted and used as features for classifying sleep stages in this study, based on the work in (Wang et al., 2023).

#### 2.4.3 Audio analysis

**FIGURE 7:**
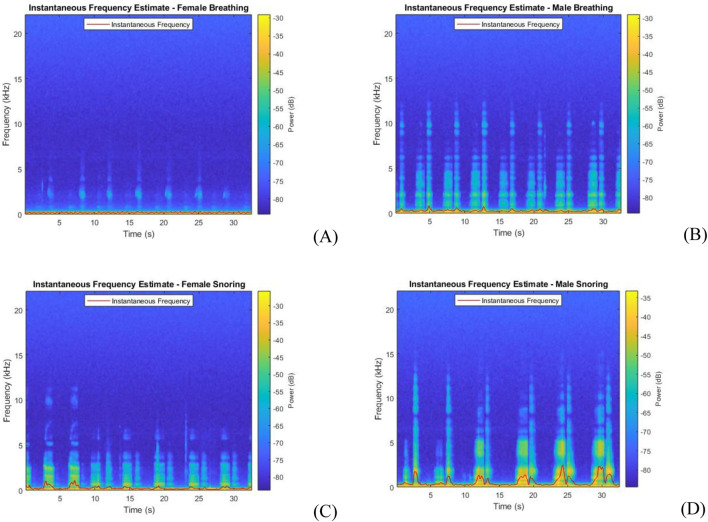
Spectrograms for the audio signal showing differences in male and female respiration during sleep cycles. **(A)** Female normal respiration, **(B)** male normal respiration, **(C)** female snoring respiration event, **(D)** male snoring respiration event.

The microphone audio was processed and analyzed based on the work in Dafna et al., 2015, Zigel et al., 2018 and incorporated with the cardiorespiratory parameters from the devices in this study. The audio signals were segmented into 30-s intervals to align with the PSG sleep stage epochs and with each sensor feature output. Each audio segment was processed with a Hanning window over 2048 samples, with 50% overlap, to reduce spectral leakage and preprocess the audio signal’s spectrogram (Jwo et al., 2019). These window sizes captured inhale/exhale sounds from respiration effectively, whereas larger window sizes resulted in diminished respiration events with this audio setup (Jaworski and Park, 2025). Each audio data frame was processed using the instantaneous frequency function in MATLAB to detect respiratory events from the audio signals, as shown in Equation 14.

$$f_{inst}\left(t\right)=\frac{\int_{0}^{\infty }f\;P\left(t,f\right)df}{\int_{0}^{\infty }P\left(t,f\right)df}$$ (14)

where *P(t, f)* is the spectrogram power spectrum with *t* and *f* denoting the time and frequency, respectively, displayed as a time-frequency distribution (Boashash, 1992a; Boashash, 1992b). The power spectrum between male and female participants was visibly different as males had louder and more low frequency power due to snoring as shown in Figure 7.

To extract respiration features from the audio signals, autocorrelation was applied to the signals as shown in Equations 15,16, [Disp-formula e16] (Box et al., 1994).

$$r_{k}=\frac{c_{k}}{c_{0}}$$ (15)

$$c_{k}=\frac{1}{T}\sum_{t=1}^{T-k}\left(y_{t}-\bar{y}\right)\left(y_{t+k}-\bar{y}\right)$$ (16)

with *y*_*t*_ and *y*_*t+k*_ being the original audio signal and the lagged audio signal by *k*, respectively, and *c*_*o*_ denoting the sample variance of the audio signal. Respiration features are extracted from the autocorrelation function, including the respiration period, intensity, and consistency, as shown in the following Equations 17–20 (Dafna et al., 2015).

$$t_{p}=peaks\left\{R_{i}\left(t\right)\right\};t_{p}=\left[t_{1},t_{2},\ldots ,t_{p}\right]$$ (17)

$${CP}_{i}=t_{1}\times Fr$$ (18)

$${CI}_{i}=R_{i}\left(t_{1}\right)$$ (19)

$${CC}_{i}=std\left\{R_{i}\left(t_{1}\right),R_{i}\left(t_{2}\right),\ldots ,R_{i}\left(t_{p}\right)\right\}$$ (20)

The positively identified peaks at the time frame from the autocorrelation audio results are represented by *t*_*p*_ *, CP*_*i*_ is the respiration cycle period that is identified by the first lagged peak excluding the zeroth lagged peak, *CI*_*i*_ is the respiration cycle intensity which is the peak amplitude and *CC*_*i*_ is the standard deviation of all the lagged peak amplitudes that indicates how periodic and consistent the respiration patterns are from the audio signals (Dafna et al., 2015).

#### 2.4.4 LSTM-TCN model

Finally, all features from each device are processed into a dataset used to train a machine learning model to classify sleep stages in 30-s epochs, and the resulting PSG sleep stages are compared. Cardiac signals acquired from the BCG were processed according to established standards for heart rate variability (HRV) analysis in the time, frequency, and nonlinear domains (Shaffer and Ginsberg, 2017). Furthermore, physiological signals are inherently nonlinear and nonstationary. Therefore, the HRV analysis was also conducted using nonlinear methods (Jaworski and Park, 2024). Features derived from all three domains were used to characterize cardiac patterns across the sleep cycle. Audio signals were processed using autocorrelation-based methods described in the previous section to extract features related to respiration cycle period, intensity, and consistency, as well as spectral energy and sound pressure level statistics representing overall acoustic intensity. Audio feature extraction followed the methodology proposed by Dafna et al. and was consistent with our prior work (Dafna et al., 2015; Jaworski and Park, 2025). In addition, CPC analysis was performed to extract features characterizing interactions between cardiac and respiratory physiological systems. The extracted CPC features included low- and high-frequency components, total power, and corresponding ratios derived from the BCG signals, consistent with features reported in the literature (Wang et al., 2023).

The processed cardiac features derived from BCG and respiratory features extracted from microphone audio represent the activity of the body’s network physiology, reflecting the coordinated interaction between the cardiovascular and respiratory systems required to maintain physiological balance during sleep. By quantifying R-depth from the BCG and respiration-related audio activity, such as normal breathing and snoring, in addition to processed heart rate variability (HRV) features, these measurements capture dynamic interactions between the cardiovascular and respiratory systems across different sleep stages. Furthermore, processing physiological signals in the nonlinear domain yields features that are closely associated with autonomic nervous system (ANS) activity, thereby emphasizing network-level physiological dynamics and improving the representation of individual sleep stages.

**FIGURE 8:**
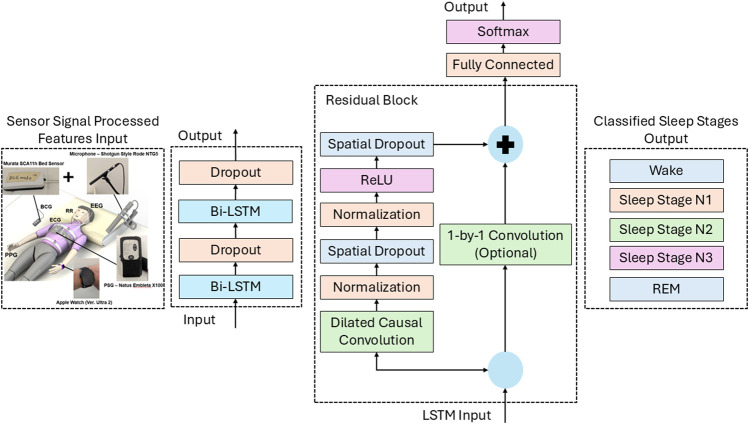
LSTM-TCN network layout of all connected layers.

The BCG + audio configuration consisted of 62 features containing the BCG-derived HRV features and the respiration-related features extracted from the microphone audio signals which was represented as an input feature vector of shape (F), where F = 62. The input batch size consisted of 5-min windows, incremented in 30-s time steps to align with the PSG sleep stage outputs for performance validation. Each 30-s label was predicted using features computed over a 5-min sliding window (updated every 30 s), yielding one feature vector per 30-s epoch. Each sleep cycle comprised approximately 400 samples of 30-s epochs, corresponding to a total duration of about 3 h per sleep cycle in this experiment. The model output was a 5-class sleep-stage (Wake, N1, N2, N3, REM) (i.e., output dimension = 5) to match the PSG output and validate the model performance. Our previous studies used a bidirectional LSTM model to classify sleep stages, while this study extends the model by combining it with a TCN model to leverage the strengths of both for improved classification. The models were trained and deployed in MATLAB, using many functions and the Deep Learning Toolbox to implement and connect all the layers. The layout of each layer of the LSTM-TCN model is shown in Figure 8.

The LSTM-TCN model combined layers from both the LSTM and TCN models to train a dataset for classifying sleep stages. First, a sequence input layer is created to initialize all the features, followed by two bi-LSTM layers with two dropout layers between them. The first bi-LSTM has 125 hidden units, and the second bi-LSTM layer has 100 hidden units, with both dropout layers set to 0.2. The TCN section of the model consists of residual blocks containing two causal and dilated 1D convolutional layers, followed by normalization, ReLU, and spatial dropout layers, which are added to the next residual block with an incremented dilation factor. The first residual block contains a 1-by-1 convolution layer that will skip this residual block when direct addition cannot be performed because the number of channels in the input and output do not match. The dilation factor is computed through Equation 21 and is based on the number of convolutional layers in the residual blocks.

$$R=\left(f-1\right)\left(2^{K}-1\right)+1$$ (21)

Within the residual function, f is the filter size that controls the receptive field size and number of learnable parameters, and K is the number of convolutional layers (Bai et al., 2018). The convolutional layer has 64 filters and 5 filter sizes, and the spatial dropout layer has a dropout factor of 0.005. The final layers include a fully connected layer and a SoftMax function layer to classify results for each sleep stage. The analyzeNetwork function in MATLAB was used to quantify the number of trainable parameters as a reference for the models’ training and classification capacity. The TCN and LSTM models contained 169k and 469.8k trainable parameters, respectively, while the combined LSTM–TCN network contained 690.8k parameters. This increased parameter count provides the combined LSTM–TCN model with greater learning and classification capacity, particularly for long-duration sleep cycles in which successive sleep stages must be retained across extended temporal dependencies. The model was trained and evaluated in a leave-one-subject-out (LOSO) cross-validation method: in each fold, all sleep cycles from one subject were held out for validation/testing, and the model was trained on the remaining subjects. This resulted in 9 folds (one held-out subject per fold), across a total of 27 sleep cycles (9 subjects × 3 sessions). To preserve temporal structure, sleep cycles were kept intact (i.e., not shuffled or concatenated across sessions) during training and evaluation. Therefore, the classifications output would reflect the natural progression of sleep stages in the sleep cycle, based on the physiological features obtained and processed by the contactless devices placed around the bed. The classified sleep stage results were compared with the PSG sleep stages that were visually scored to validate the effectiveness of the entire system.

## 3 Results

The sleep stage classifications produced by the combined LSTM-TCN model, as well as those of the LSTM and TCN models individually for comparison, are shown in Table 1. Model performance was evaluated across all sleep stages—wake, N1, N2, N3, and REM—with outputs generated according to the AASM guidelines and PSG results. For further analysis, sleep stages were also relabeled into three classes by combining N1, N2, and N3 into non-REM, while maintaining wake and REM as distinct stages. As this system does not include the full range of sensors that a PSG device does, it may be challenging for a non-intrusive system to identify all the nuanced sleep stages from cardiorespiratory signals obtained from a BCG and a microphone alone. The combined LSTM-TCN network achieved the highest accuracy of 80.51% agreement with the PSG sleep stages when relabeled as wake/REM/non-REM, outperforming the individual models. This primary performance was obtained using the core contactless configuration (BCG + Audio).

**TABLE 1:** Accuracy and Cohen’s Kappa with standard deviation of cross validation fold metrics for the LSTM-TCN model and LSTM and TCN models separately for all sleep stages (wake, N1, N2, N3, REM) and relabeled wake, REM, and non-REM sleep stages.

	All stages		REM/non-REM	
	Accuracy	Kappa	Accuracy	Kappa
LSTM-TCN	63.24 ± 4.39	0.50 ± 0.04	80.51 ± 2.74	0.65 ± 0.06
TCN	61.90 ± 7.43	0.48 ± 0.08	74.37 ± 5.82	0.56 ± 0.09
LSTM	54.59 ± 2.41	0.36 ± 0.02	66.61 ± 6.11	0.43 ± 0.07

**FIGURE 9:**
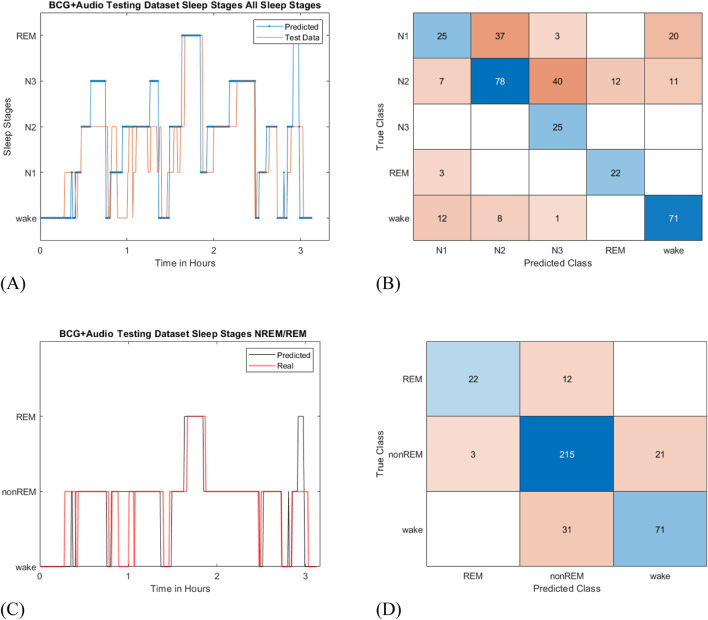
BCG + audio classified sleep stages from the LSTM-TCN model and the corresponding confusion matrix for all stages and wake/REM/non-REM. **(A)** is the hypnogram for all sleep stages, and **(B)** is the corresponding confusion matrix. **(C)** and **(D)** are the hypnogram for the relabeled sleep stages (wake/REM/non-REM) and the corresponding confusion matrix, respectively.

The sleep stages classified by the LSTM-TCN model, compared with the PSG output sleep stages, are shown in Figure 9, along with the corresponding confusion matrix. The statistical measures for each classified sleep stage from each model are shown in Tables 2–6. These tables show the sensitivity, specificity, precision, and F1 score for correctly classified sleep stages from the sleep system in this experiment, compared to the PSG sleep stages. The model accurately classified instances of sleep and wake; however, it exhibited difficulty distinguishing between the deeper (N2 and N3) sleep stages among all the other sleep stages. As N2 sleep comprises most of a typical sleep cycle, misclassifying different sleep stages would affect N2 sleep stages the most, yet the model showed balanced sensitivity and specificity. While wake stages were uniquely identifiable due to the dynamic physiological signals associated with increased activity. Short sleep durations resulted in few REM stages, with high sensitivity but low specificity due to misclassifying other stages as REM. The wake, N1, and N2 sleep stages generally demonstrated robust sensitivity and specificity, indicating reliable classification performance. In contrast, the deeper sleep stages, N3 and REM, were more difficult to classify from a cardiorespiratory perspective across all models. Transitions between non-REM sleep stages may be subtle, as the body relaxes and decreases heart and respiratory rates during deeper sleep, further complicating classification. Nevertheless, the TCN model and LSTM-TCN model demonstrated improved identification of sleep and wake instances throughout the sleep cycle, particularly under the BCG + audio (contactless) configuration.

**TABLE 2:** Statistical measures for classified wake stages.

	Wake				
	Sensitivity	Specificity	Precision	F1	P-value
LSTM-TCN	0.91 ± 0.04	0.86 ± 0.07	0.93 ± 0.002	0.92 ± 0.02	0.242
TCN	0.92 ± 0.02	0.74 ± 0.16	0.83 ± 0.07	0.87 ± 0.03	<0.001
LSTM	0.89 ± 0.03	0.76 ± 0.08	0.86 ± 0.04	0.87 ± 0.02	0.031

**TABLE 3:** Statistical measures for classified N1 stages.

	N1				
	Sensitivity	Specificity	Precision	F1	P-value
LSTM-TCN	0.89 ± 0.05	0.61 ± 0.05	0.97 ± 0.02	0.93 ± 0.03	<0.001
TCN	0.87 ± 0.07	0.31 ± 0.17	0.97 ± 0.02	0.92 ± 0.05	<0.001
LSTM	0.88 ± 0.07	0.34 ± 0.01	0.97 ± 0.03	0.92 ± 0.05	<0.001

**TABLE 4:** Statistical measures for classified N2 stages.

	N2				
	Sensitivity	Specificity	Precision	F1	P-value
LSTM-TCN	0.81 ± 0.05	0.51 ± 0.14	0.85 ± 0.01	0.83 ± 0.03	0.011
TCN	0.82 ± 0.09	0.55 ± 0.14	0.84 ± 0.05	0.82 ± 0.03	<0.001
LSTM	0.79 ± 0.03	0.38 ± 0.14	0.75 ± 0.07	0.77 ± 0.05	0.01

**TABLE 5:** Statistical measures for classified N3 stages.

	N3				
	Sensitivity	Specificity	Precision	F1	P-value
LSTM-TCN	0.96 ± 0.05	0.48 ± 0.17	0.86 ± 0.07	0.90 ± 0.06	<0.001
TCN	0.94 ± 0.05	0.7 ± 0.24	0.95 ± 0.04	0.94 ± 0.01	<0.001
LSTM	0.94 ± 0.05	0.53 ± 0.38	0.98 ± 0.02	0.94 ± 0.02	0.01

**TABLE 6:** Statistical measures for classified REM stages.

	REM				
	Sensitivity	Specificity	Precision	F1	P-value
LSTM-TCN	0.96 ± 0.04	0.34 ± 0.13	0.92 ± 0.03	0.94 ± 0.02	<0.001
TCN	0.97 ± 0.03	0.29 ± 0.2	0.91 ± 0.03	0.94 ± 0.03	<0.001
LSTM	0.95 ± 0.06	0.21 ± 0.1	0.83 ± 0.03	0.89 ± 0.03	0.075

## 4 Discussion

This study utilized contactless devices functioning together in a system to evaluate sleep stages as a convenient alternative to conventional sleep studies, which typically rely on PSG and require attendance at a sleep clinic. The BCG sensor and microphone captured cardiorespiratory signals, from which features were extracted to classify sleep stages. The sleep stage classification results demonstrated improved accuracy when using the proposed LSTM–TCN model. The combined model correctly classified wake/REM/non-REM stages at about 80.51% accuracy (Cohen’s κ = 0.65) across all participants in the dataset for the BCG + audio configuration. Separately, the LSTM and TCN models performed adequately, with accuracies of 66.61% (κ = 0.43) and 74.37% (κ = 0.56), respectively, for the BCG and audio configuration with wake/REM/non-REM sleep stage labels. The TCN model outperformed the LSTM model by capturing both short- and long-term dependencies, especially since a sleep cycle spans multiple hours while sleep stages change in 30-s intervals according to analysis standards (Biswas et al., 2025). Although LSTM models can be effective for long-term modeling, their performance may decline in later portions of the sleep cycle due to the influence of the long-term memory (Rahda et al., 2019). Combining both networks would leverage the strengths of each by learning the long-term patterns of the entire sleep cycle from the LSTM network, while the TCN would focus on short-term changes within the 30-s epoch.

The statistical measures presented in Tables 2–6 illustrate the classification performance of each sleep stage from the model compared to the PSG outputs. The sensitivity and specificity showed generally good spread across wake, N1, N2, sleep stages in the LSTM-TCN model, while N3 and REM were overly sensitive in the classification. The wake stages were highly sensitive and specific because they mostly occurred with movement activity as the participant entered/exited the bed and moved around to become comfortable before falling asleep. They provided distinguishable features to identify wake or sleep instances. N1 sleep stages had few occurrences in the short sleep sessions but were distinguishable from other sleep stages due to generally occurring around wake stages as the participant transitioned from wake into sleep. N2 sleep comprises most of a sleep cycle and may cause the model to assume that most epochs fall into this stage, resulting in misclassifications of the other stages, as shown by the 81% sensitivity and 51% specificity in the LSTM-TCN model. The N3 stage seemed to be the most difficult to classify, as the difference between N2 and N3 from a cardiorespiratory perspective may be subtle and not easily distinguishable, leading to misclassification between these stages. Overall, classifying all sleep stages with non-intrusive devices yielded an average accuracy of approximately 55%–63% across all models (Table 1). Further feature processing or the inclusion of additional sensors may enhance classification performance.

This study expands on our previous experiment evaluating sleep quality with non-intrusive devices, including a high-quality microphone for audio signal acquisition (Jaworski and Park, 2025). A key advancement in the present study is the diversification of the dataset by recruiting 9 participants, with an approximately equal split between males and females each sleeping for 3 sessions. In contrast, the previous study involved a single participant sleeping across multiple sessions and focused on validating the integration of an audio microphone within an IoT sleep quality evaluation system. In the present study, female participants generally exhibited lower respiratory sound power than males, as reflected in the spectrograms in Figure 7, while snoring was more prevalent among males with increased low-frequency power (Dosman et al., 2022). Although audio recorder and microphone settings were kept constant across participants, these findings suggest that participant-specific adjustment of audio settings may improve measurement accuracy. Moreover, reduced audio quality in participants who moved away from the microphone during sleep highlights the importance of sensor placement.

Cardiorespiratory measurements from the BCG were analyzed to obtain additional features from CPC to classify sleep stages. The features included in this analysis were related to the power derived from the low and high frequency portions of the CPC and their ratio based on Wang et al. (2023). The low and high frequency powers obtained from the BCG signals showed increased CPC power ratio during wake and lighter instances of sleep. The inclusion of a comprehensive set of cardiorespiratory features derived from BCG and microphone audio provided a broad range of information for sleep stage analysis by capturing physiological interactions between the cardiovascular and respiratory systems. Slower heart rate and respiration rate are characteristic of different sleep stages, and analyzing HRV features in conjunction with respiration-related audio features enables assessment of how these physiological systems interact and adapt during sleep. These coordinated responses reflect the underlying physiological networks and contribute to the identification of the participant’s current sleep stage. Overall, these findings support contactless inference of sleep-stage–dependent changes in cardiorespiratory coordination (e.g., CPC- and HRV-derived coupling features), consistent with network physiology perspectives on coupled subsystem dynamics during sleep.

Extra noise from movements can create artefacts in cardiorespiratory activity measured with a BCG, as the extra vibrations can distort the signal and increase power during more active sleep states. Furthermore, measuring cardiorespiratory signals through vibrations with the BCG can cause less distinct J-peaks that result in relatively higher errors compared to the R-peaks obtained from an ECG (Park et al., 2018). CPC features are valuable because they can be used to evaluate sleep disorders such as insomnia, hypertension, and sleep apnea in addition to aiding sleep stage classification by including more diverse features (Ashry et al., 2021). This study involved only one participant in bed at a time to ensure that all device measurements were attributable to a single individual, thereby enabling precise analysis of sleep quality. Accommodating multiple people in the same bed would require redesigning the device setup to isolate measurements for each person, as well as implementing additional signal-processing methods to mitigate artefacts introduced by the presence and movements of another sleeper.

## 5 Limitations and future work

The main limitation of this study is the short duration of sleep cycles, with at least 3 h per session, even though participants were able to enter all sleep stages. Future studies should examine longer sleep sessions so participants can fully enter all sleep stages, providing more diverse data than our prior single-participant study for training. This study expanded on our previous work by including a more diverse group of participants and provided valuable insight into the future direction the experiments could take by targeting specific demographics with tuned parameters and classification models. However, a dataset with more participants would also allow us to more robustly generalize the performance of the models and have a more reliable analysis, including tighter confidence intervals and more stable estimates under LOSO evaluation, with future studies focusing on collecting data from more participants. In this study, only a single participant slept in the bed at a time. However, because it is common for couples to share a bed, extending the system to support multiple occupants would enhance its practical applicability. Future work will focus on recruiting specific demographic groups, exploring common sleeping arrangements such as multiple individuals sharing a bed, and evaluating alternative feature-processing strategies and tuned machine learning parameters to improve system performance under these conditions.

## 6 Conclusion

This study integrated non-intrusive devices in a contactless environment using BCG and a microphone to collect physiological data for sleep stage classification. Using the BCG and microphone with the combined LSTM-TCN model has the advantage of each device’s contactless nature, allowing physiological signals to be collected from participants, while they sleep naturally, without any interference from medical equipment placed on their bodies. Natural sleep measurements can provide accurate data for a machine learning model to classify sleep stages, yielding results comparable to a gold-standard PSG system. Processing features using HRV methods and emphasizing nonlinear analysis to capture the dynamics of physiological signals demonstrated that the LSTM-TCN model effectively classified sleep stages. In addition, measuring cardiorespiratory parameters from each device within a contactless system enables characterization of interactions between physiological systems as they work together across different stages of sleep. Deviations from baseline physiological regulation may indicate disruptions in network physiology and be associated with sleep-related or broader health disorders, potentially warranting further clinical evaluation. The non-intrusive nature of such a system enables longitudinal, in-home monitoring of physiological and sleep data, allowing clinicians to evaluate trends that may warrant a comprehensive clinical sleep study.

## Data Availability

The raw data supporting the conclusions of this article will be made available by the authors, without undue reservation.
